# Effect of Larvae Treated with Mixed Biopesticide *Bacillus thuringiensis -* Abamectin on Sex Pheromone Communication System in Cotton Bollworm, *Helicoverpa armigera*

**DOI:** 10.1371/journal.pone.0068756

**Published:** 2013-07-09

**Authors:** Li-Ze Shen, Peng-Zhou Chen, Zhi-Hong Xu, Jian-Yu Deng, Marvin-K Harris, Ruchuon Wanna, Fu-Min Wang, Guo-Xin Zhou, Zhang-Liang Yao

**Affiliations:** 1 Department of Plant Protection, School of Agriculture and Food Science, Zhejiang Agriculture and Forestry University, Lin’an, Zhejiang, China; 2 Department of Entomology, Texas A&M University, College Station, Texas, United States of America; 3 Department of Agriculture Technology, Mahasarakham University, Kantarawichai District, Maha Sarakham, Thailand; University of Tennessee, United States of America

## Abstract

Third instar larvae of the cotton bollworm (*Helicoverpa armigera*) were reared with artificial diet containing a *Bacillus thuringiensis* - abamectin (BtA) biopesticide mixture that resulted in 20% mortality (LD_20_). The adult male survivors from larvae treated with BtA exhibited a higher percentage of “orientation” than control males but lower percentages of “approaching” and “landing” in wind tunnel bioassays. Adult female survivors from larvae treated with BtA produced higher sex pheromone titers and displayed a lower calling percentage than control females. The ratio of Z-11-hexadecenal (Z11–16:Ald) and Z-9-hexadecenal (Z9–16:Ald) in BtA-treated females changed and coefficients of variation (CV) of Z11–16:Ald and Z9–16:Ald were expanded compared to control females. The peak circadian calling time of BtA-treated females occurred later than that of control females. In mating choice experiment, both control males and BtA-treated males preferred to mate with control females and a portion of the Bt-A treated males did not mate whereas all control males did. Our Data support that treatment of larvae with BtA had an effect on the sex pheromone communication system in surviving *H.armigera* moths that may contribute to assortative mating.

## Introduction

Pesticides provide a primary and non-substitutable method to control pests so that crop yield potential is conserved [1]. However, pesticide abuse is a serious problem all over the world and pesticide residue in fields can increase risks of threats to human health, inducing pesticide resistance, and killing of non target species [2], [3].

There are many studies that focus on the sublethal effects of pesticides on target pests and beneficial arthropods. A median lethal dose (LD_50_) or lethal concentration (LC_50_) estimate has been the classical laboratory method for assessing the side effects of pesticides on beneficial arthropods for decades but do not specifically address sublethal effects [2].

Many researchers have demonstrated that pesticides impact natural enemies directly or indirectly [4]–[11]. The longevity of *Microplitis mediator*, a parasitoid of the cotton bollworm (*Helicoverpa armigera*) decreased significantly after being fed with 10% honey water containing a sublethal dose of a *Bacillus thuringiensis* and abamectin (BtA) [10].

Studies of the sublethal effects of pesticides have also included work on agricultural pests that examined side effects on the sex pheromone and chemical communication systems of Lepidoptera. Sublethal permethrin treatment reduced the incidence of calling behavior of female pink bollworm (*Pectinophora gossypiella*) [12] and activation (wing fanning) of male survivors to sex pheromone [13]; similar results were obtained for cabbage looper (*Trichoplusia ni*) female moths [14]. However, treatment of oriental fruit male moths (*Cydia molesta*) with octopamine induced hypersensitivity to olfactory signals [15], as did chlordimeform treatment of male *T.ni* [16]. A sublethal (LD_1_) dose of chlordimeform stimulated pheromone emission early in scotophase and exhibited a high percentage of calling behavior in treated *T.ni* moths [17]. However, female Asian corn borer (*Ostrinia furnacalis*) moths decreased sex pheromone titers and pheromone biosynthesis activating neuropeptide (PBAN)-like activity following treatment with deltamethrin [18].

All these studies were focused on sublethal effects of pesticides on the adult stage of lepidopteron moths. Since 2003, some researchers began to focus on adult survivors from larvae treated with a sublethal dose of pesticide and pests with high resistance to pesticides. Adults of *O.furnacalis* that survived treatment with deltamethrin during the first and third instars produced higher titers of sex pheromone, expanded coefficients of variation of the ratio of sex pheromones and displayed a lower response to sex pheromone in wind tunnel testing [19]. Tobacco cutworm moths (*Spodoptera litura*) emerging from larvae treated with a sublethal dose of deltamethrin performed similarly [20]. Male moths of *H.armigera* treated with Bt from the third larval instars showed higher EAG responses to sex pheromones than controls [21], as did tebufenozide and abamectin resistant diamondback (*Plutella xylostella*) male moths [22]. However, *P. xylostella* moths elicited similar EAG responses after exposure to indoxacarb compared with controls [23].

Biopesticide usage is increasing every year, even more with the prespective that mixtures of pesticides are theoretically more effective in delaying resistance than alternating usage of pesticides [24]. An example of this strategy is the conjugation of the toxins from *Bacillus thuringiensis* with the toxin of abamectin, to form a new biopesticide called BtA [25]. This BtA mixture has been widely used to control agricultural pests [26]–[28] and the usage of BtA was in the hundreds of tons in several provinces of China in 2011. Despite this heavy use, how this biopesticide mixture affects both pests and natural enemies warrants further study. Effects of BtA on development of cotton bollworm and longevity of adult *M.mediator* parasitoids has begun to be studied [10]. In this study, we continue to focus on effects of BtA on the sex pheromone communication system of adult survivors of *H.armigera* obtained from 3^rd^ instar larvae reared on artificial diet containing sublethal doses of BtA. Although the individual effects of these two biopesticides on sex pheromone communication systems has been studied [22], [29], additional work is needed to clarify the effects of the biopesticide mixture on the sex pheromone communication system. In this study, we evaluate the effects of BtA on calling behavior, production of sex pheromone in *H.armigera* females, the behavior responses of males to pheromone lures in a wind tunnel and mating choice in mating cages.

## Methods and Materials

### Insect

Larvae of *H.armigera* obtained from the laboratory of Zhejiang Academy of Agricultural Sciences (China) were reared at 25±1°C, 75% relative humidity (r.h.), and L14:D10 photoperiod on artificial diet [30] in the insectary. They were separated into two experimental populations, one was reared on a standard artificial diet without any pesticides, while the other was reared on an artificial diet containing a sublethal dose of BtA applied during the 3^rd^ larval instar. Larvae were first reared in groups in plastic casing (20 cm×30 cm×7 cm) with artificial diet until the 3^rd^ instar, when they were transferred to and reared singly in plastic petri dishes (6 cm diam×2 cm depth) where fresh diet was provided every 4 days. Pupae were separated by sex, and male and female moths were held in glass test tubes (3 cm diam×10 cm high) with 10% honey water.

### Pesticides and Sex Pheromones

The biopesticide BtA was tested, was composed of *B.thuringiensis* var.kurstaki (16,000 IU/mg, Fujiang Pucheng Green Shell Biological Technology, Pucheng, China) and Abamectin (0.18%EC, Zhejiang Shenghua Biok Biological, Zhejiang, China) mixed by Sendebao Bioproducts (Wenzhou, China). Two sex pheromone components Z-11-hexadecenal (Z11–16:Ald) and Z-9-hexadecenal (Z9–16:Ald) were obtained commercially (Shin-Etsu Chemical Co. Ltd., Japan). The purity of these two pheromones was detected to be more than 95% by Gas Chromatography (GC) analysis.

### Effects of BtA on Development from 3^rd^ Instar Larvae until Eclosion in *H.armigera*

The method for this experiment was similar to that of Wanna et al. (2010) [10]. The sublethal BtA concentrations tested in the artificial diet were 0.5, 1, 2, 4 and 8 µg/g. The stock solutions were prepared by first dissolving BtA in distilled water and then adding it to the liquid diet at a temperature of 50°C before solidification. Neonate larvae were reared in plastic casings (20 cm×30 cm×7 cm) with standard artificial diet and then treatments were established by transferring single 3^rd^ instar larvae into plastic petri dishes (6 cm diam×2 cm high) provisioned with fresh artificial diet with different concentrations of BtA every 4 days until pupation. There were three replications for each concentration and 100 larvae per replication. Data on larval mortality, pupation rate, abnormal pupa rate, male pupa weight and female pupa weight were obtained at the end of the experiment. The surviving moths were held in glass tubes (3 cm diam×10 cm high) with 10% honey water and used in subsequent experiments.

### Calling Behavior

3-day-old virgin female moths were individually transferred into a glass test tube (3 cm diam×10 cm high) with 10% honey water and put into a darkroom (25±1°C, 75% r.h., and L14:D10 photoperiod). Calling behaviors were observed by eye with a dim incandescent red backlight at 30 min intervals throughout the 10-h scotophase in entirely dark environmental conditions. Each female was scored as either calling or not calling. There were three replications for control females and BtA-treated females and 10 females per replication. Calling behavior characteristics observed were upward abdomen, wide spread wings with fanning, and ovipositor or gland visually evident protruding from the end of abdomen under red light (0.3lux).

### Gas Chromatography Analysis

**Figure 1:**
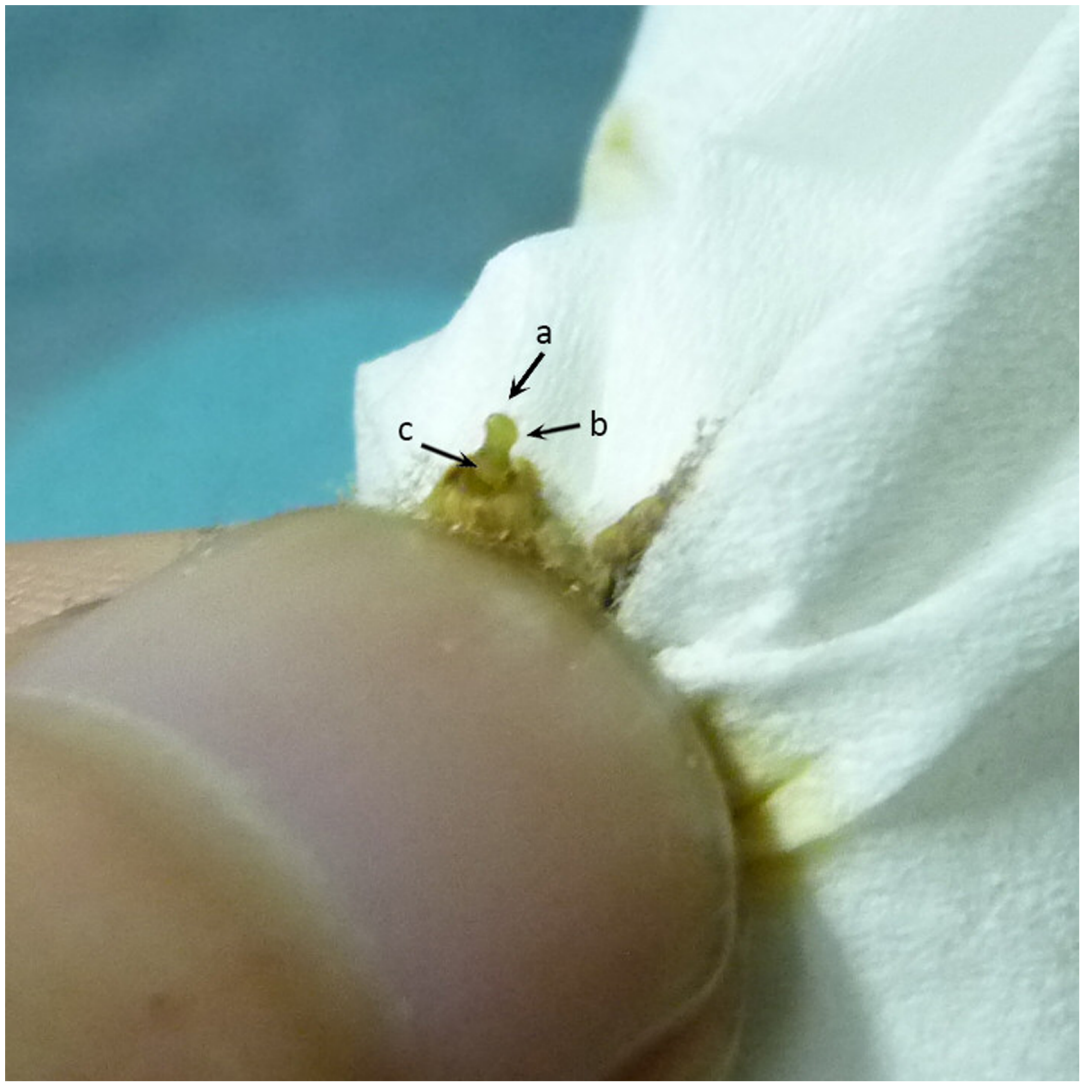
Excision of *H.armigera* sex pheromone gland for being extractioned. (a) ovipositor; (b) gland; (c) cuticle.

The ovipositors and associated sex pheromone glands were excised from 3-day-old virgin female moths during their peak calling period (7 h∼8 h after the initiation of scotophase). Glands were forced out by squeezing the abdomen and excising the tip at the 8∼9^th^ abdominal segment with ophthalmic scissors (**Figure 1**). Each gland was soaked in 10 µL redistilled hexane for 30 min to extract pheromone components. Then, the ovipositors and associated sex pheromone glands were removed and the extracts were maintained at 0°C until GC analysis by using standard methods. The GC-standards (GCS) of Z11–16:Ald and Z9–16:Ald in hexane were detected by GC first with concentrations of 25, 50, 100, 200 and 400 ng/µL. The injection volume was set at 1 µL. Standard curves were drawn by calculating the peak areas of GCS. The extracts were concentrated to 2 µL, and then the concentrated extracts were injected into a GC (GC-2010, Shimadzu, Japan) equipped with a flame ionization detector (FID), a split/splitless injector, and a fused silica capillary column (RTX-5, 30 m×0.25 mm ID, 0.25 µm film thickness, SGE, Australia) in splitless mode. Nitrogen was used as the carrier gas. The oven temperature was maintained at 100°C for 2 min, and programmed at 10°C /min to 200°C. The injector and FID temperature were set at 220°C and 250°C. The quantity of each component was calculated based on the peak area, and calibrated by comparing it with standard curves of Z11–16:Ald and Z9–16:Ald. 22 glands of both control females and BtA-treated females were extracted and tested.

### Wind Tunnel Bioassay

**Figure 2:**
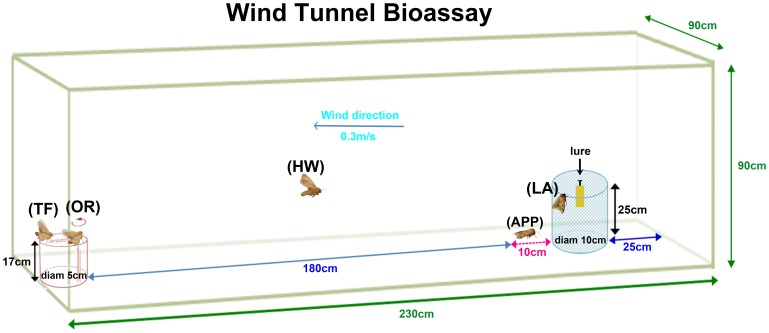
Five parameters used in the wind tunnel bioassay: take flight (TF), orientation (OR), half up-wind to lure (HW), approaching the cage containing the lure (within 10cm) (APP), landing on the cage containing the lure (LA).

The methods for wind tunnel tests were similar to Deng et al. (2004) [31]. The tests were performed in a Plexiglas wind tunnel, 230 cm×90 cm×90 cm under conditions of 25±1°C, 75r.h., 0.3lux (red light), and air speed of 0.3 m/s. Naïve male moths were tested at the 3^rd^ scotophase by exposure to sex pheromone. Before the onset of scotophase, both control male moths and adult male survivors from larvae treated with BtA were transferred individually into glass test tubes (3 cm diam×10 cm high). All tubes were held until 7^th^ hr into scotophase. Moths were allowed to acclimate to tunnel conditions for 60 min, and then introduced into the tunnel individually. Lure (filter paper) containing 300 ng of the 97∶3 blend of Z11–16:Ald and Z9–16:Ald was pinned on a 25 cm high iron shelf placed on the midline of the wind tunnel and 25 cm away from the up-wind end. The iron shelf with lure was packaged with a cylindrical wire netting cage (10 cm diam×25 cm high). Male moths were introduced into the wind tunnel by using an open-ended release cage (5 cm diam × 10 cm high) placed 17 cm high and 200 cm from the lure. Each male was allowed 2 min to respond and scored for the following behaviors: taking flight (TF), orientation flight (OR), half up-wind to lure(HW), approaching the cage containing the lure(within 10 cm) (APP), landing on the cage containing the lure (LA) (**Figure 2**). 69 contral males and 81 BtA-treated males were tested. Each male was tested only once.

### Mating Choice Experiment

**Figure 3:**
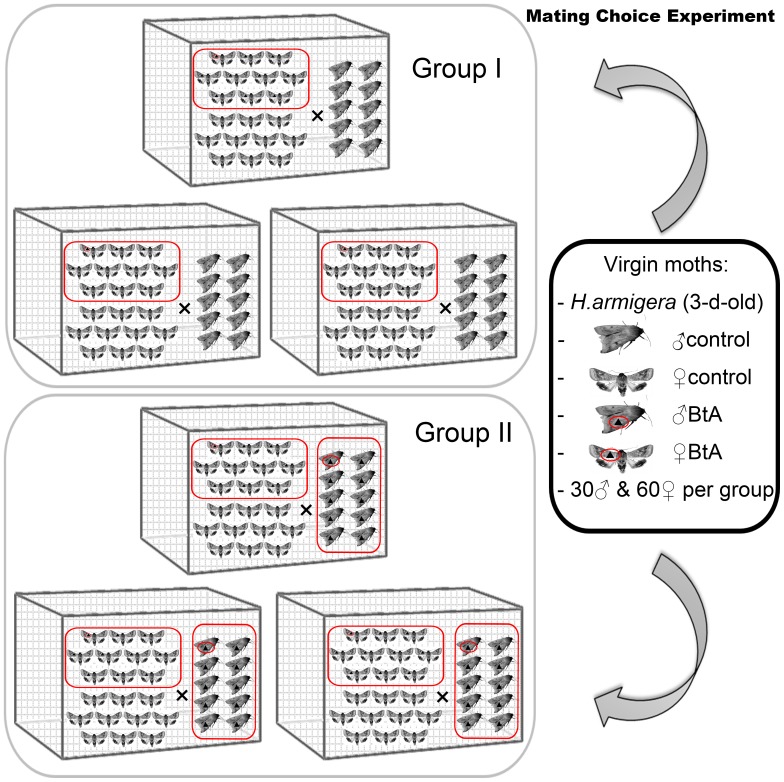
Mating choice experiment of *Helicoverpa armigera* with two groups.

3-day-old virgin moths were used in this experiment. One group consisted of 10 control males and 10 BtA-treated and control females. The other group was 10 BtA-treated males and 10 BtA-treated and control females. These moths were introduced into mating cages (120 cm × 80 cm × 80 cm) before scotophase. Both BtA-treated males and females were marked on their wings with carbon black ink (this does not interfere with males’ mating behavior). Three replicates were performed for each group (**Figure 3**). Observations were made at 1 h intervals throughout the 10 h scotophase by using red light (0.3lux). Male moths were removed if mated. Experiments were tested in darkroom under conditions of 25±1°C, 75r.h. and 10 h scotophase in entirely dark environmental conditions.

### Statistics

Data analysis for development of *H.armigera* was performed using LSD test (P<0.05). Statistical comparisons of percentage of calling behaviors and female mating percentages between control moths and moth survivors from the larval treatment with BtA used the student’s t test. The distribution of male moth responses to lures in the wind tunnel bioassay used a χ^2^ test. Mean titers of sex pheromones between control females and BtA-treated females were analyzed using a nonparametric test (Mann-Whitney U test). Percentage data were arcsine square root transformed before analysis. All data were analyzed by IBM Statistics SPSS 19.0 and DPS 9.5 [32].

## Results

### Effects of BtA on Development of *H.armigera* from 3^rd^ Instar Larva to Eclosion

Treatment with BtA significantly affected the development of *H.armigera* (**Table 1**). The mortality of 3^rd^ instars after feeding with BtA diet was significantly higher (P<0.05) than the control. In our assays, a BtA concentration of 2 µg/g resulted in 20% mortality and subsequent experimentation was done with this concentration compared to control. The means of pupation rate, normal pupa rate and male pupal weight of larvae treated with treatment from 0.5 µg/g to 8 µg/g of BtA were significantly lower (P<0.05) in controls, but the mean of female pupal weight was significantly higher (P<0.05) in BtA-treated compared to controls. Also, male and female mean pupal stages were delayed with larval treatment from 0.5 µg/g to 8 µg/g and most pupae were in a diapause condition (four eye spots in line observed on the compound eye).

**Table 1:** Effects of BtA on development of *Helicoverpa armigera* from 3^rd^ larvae to eclosion (Mean ± SEM)^a^ N = 100*3.

BtA concen- tration (µg/g)	3^rd^ instar larvae mortality (%)	Pupation rate (%)^b1^	normal pupa rate (%)^c^	male pupal weight (mg)^b2^	No. of normal male pupas	Male pupal stage (day)^d1^	female pupal weight (mg)^b3^	No. of normal female pupas	Female pupal stage (day)^d2^
control	1.33±0.67a	98.98±0.59b	99.33±1.15e	250.12±2.14de	48.33±1.45e	10.60±0.09a	239.71±1.99c	48.33±0.88e	11.09±0.12a
0.5	9.33±2.85b	91.12±0.92b	82.61±2.13d	252.51±2.61e	38.67±5.67d	15.11±0.14b	246.56±2.67d	29.67±3.48d	16.78±0.19b
1	12.00±1.53b	82.71±5.29a	80.73±2.09d	243.37±2.81d	29.33±1.45c	16.85±0.12c	264.39±2.41e	29.33±2.67d	17.10±0.15b
2	20.67±2.33c	81.94±0.92a	56.49±3.70c	231.17±2.88c	16.67±1.20b	17.14±0.16c	259.10±3.02e	20.00±0.00c	17.10±0.21b
4	26.00±1.53c	79.83±2.72a	43.46±2.28b	214.44±4.28b	13.33±0.67ab	17.17±0.17c	226.89±5.84b	12.33±0.88b	16.97±0.29b
8	47.67±3.18d	80.44±2.53a	31.62±2.58a	191.22±7.39a	7.33±1.86a	17.09±0.29c	201.67±6.94a	6.00±1.15a	17.10±0.41b

a Means within the same column followed by the same letter are no significantly different (LSD test: P>0.05).

b Including normal pupas and malformed pupas.

c Only including normal pupas, total numbers of pupas = No. of male normal pupas+No. of female normal pupas.

d Only including pupas that moths could emergence from successfully.

### Calling Behavior of Females

**Figure 4:**
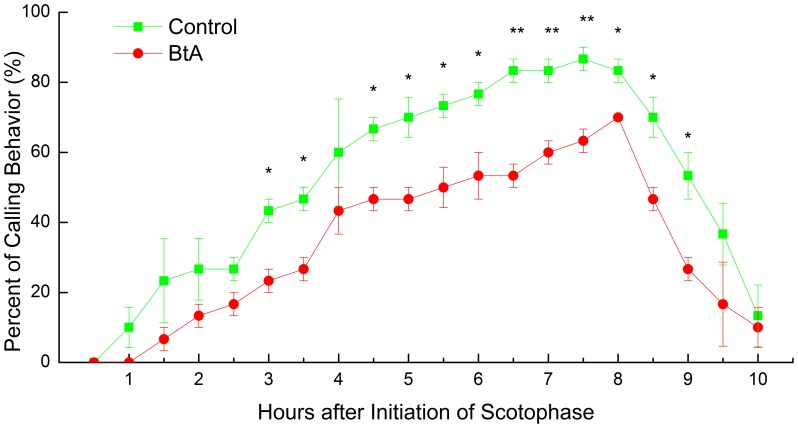
Calling percentages of female moths. The moths were recorded as calling or not calling during a 10-h scotophase after treating 3rd instar larvae with BtA artificial diet (N = 30) or standard artificial diet (N = 30). The data (Mean±SEM) marked with “*” and “**” are significantly different at P<0.05 and P<0.01 based on student’s t test after mean percentages were arsine square-root transformed.

The periodicity of calling behaviors by females that survived treatment as 3^rd^ instar larvae with BtA was similar to that of the control (**Figure 4**). Control females started calling after 1 h into the 10-h scotophase, but BtA-treated females started 0.5 h later (**Figure 4**). From 0.5 h to 2.5 h and 8 h to 9 h, there were no significant differences in percentages of calling between control females and female survivors (**Figure 4**). From 3 h to 6 h and 9.5 h to 10 h, calling percentages of control females were significantly higher than female survivors (P<0.05) except at 4 h (**Figure 4**).Compared with BtA-treated females, calling percentages of control females were significantly higher (P<0.01) during the calling peak period (6.5 h to 8 h) (**Figure 4**). Calling peak time of BtA-treated females was at 8 h, but calling percentage of control females was decreased from their peak at this time. After calling peak periods, both calling percentages had decreased sharply but there were still some females that kept calling after scotophase ended.

### GC Analysis of Female Sex Pheromones

The mean titers of sex pheromones from control females conformed to a normal distribution but BtA-treated females did not. These data were analyzed using a nonparametric test (Mann-Whitney U test). The BtA-treated females produced significantly higher (P<0.01) amounts of Z11–16:Ald and Z9–16:Ald compared to control females (**Table 2**). The mean blend ratio of Z11–16:Ald and Z9–16:Ald in BtA-treated females was 5.80±0.28 and significantly different (P<0.01) from that of control females (9.76±0.16) (**Table 2**). In addition, coefficient of variations (CV) in Z11–16:Ald, Z9–16:Ald and the sum of BtA-treated females were 52.57%, 44.74% and 50.77%, significantly higher (P<0.01) than that of control females (10.62%, 12.55% and 10.54%) (**Table 2**).

**Table 2:** Mean pheromone titer(ng)±SEM (CV%) and blend ratio in BtA and control females of *H.armigera* (N = 22).

Treatment	Mean Titer(ng) ± SEM (CV%)^a^			ratio^b^
	Z11–16:Ald	Z9–16:Ald	Sum	
Control	47.73±1.08(10.62)	4.92±0.13(12.55)	52.64±1.18(10.54)	9.76±0.16**
BtA	166.45±19.10(52.57)**	28.76±2.81(44.74)**	195.21±21.62(50.77)**	5.80±0.28

a The data marked with “**” are significantly different at P<0.01 by using a nonparametric test (Mann-Whitney U test). CV = SD/Mean.

b The blend ratio is Z11–16:Ald/Z9–16:Ald.

### Wind Tunnel Bioassay

**Figure 5:**
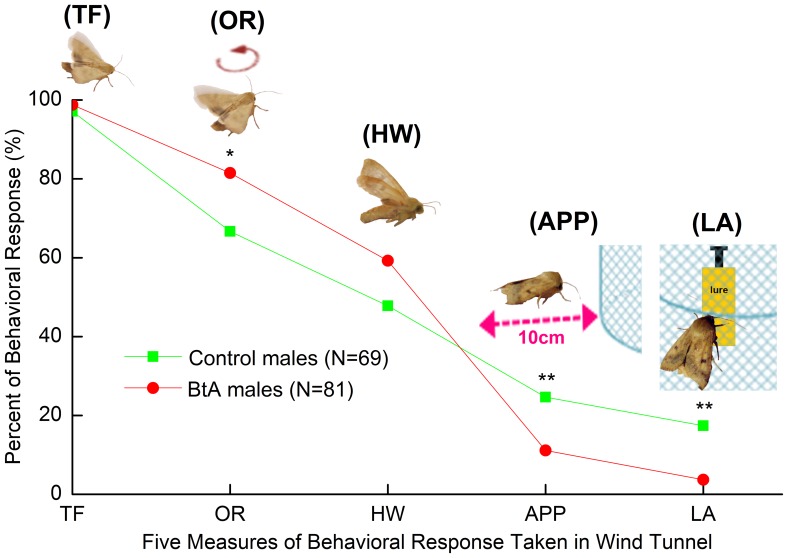
Behavioral responses of male moths to a pheromone lure in the wind tunnel bioassays. In lure (filter paper), the dosage of sex pheromones was 300 ng with the 97∶3 blend of Z11–16:Ald and Z9–16:Ald. Five parameters taken in the wind tunnel: TF = taking flight, OR = orientation flight, HW = half up-wind to lure, APP = approaching the cage containing the lure (within 10 cm), LA = landing on the cage containing the lure. The data marked with “*” and “**” are significantly different at P<0.05 and P<0.01 as shown by a χ^2^ test.

Compared with control males, the behavioral responses of male survivors from 3^rd^ instar larvae treated with BtA were similar in “TF” and “HW” behavior, however, the percentage of BtA-treated males was significantly higher (P<0.05) than control males in “OR” behavior (**Figure 5**). There were significant differences (P<0.01) between BtA-treated males and control males in behaviors of “APP” and “LA” and percentages of BtA-treated males approaching to the cage and landing on the cage containing the lure were significantly lower (P<0.01) than in controls (**Figure 5**).

### Mating Choice

**Figure 6:**
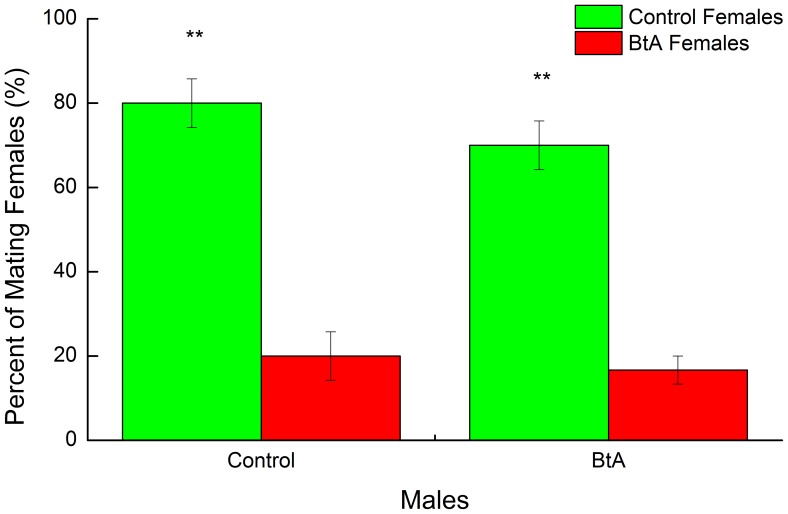
Percentages of females that mated with control males and BtA females. The bars (Mean±SEM) marked with “**” are significantly different at P<0.01 by using Student’s t test after percentages were arsine root square transformed.

Results from two non-random mating choice experiments showed that control and BtA-treated males preferred to mate with control females. Percentages of mating control females with control males and BtA-treated males were both significantly higher (P<0.01) than for BtA-treated females (**Figure 6**). The percentage of unmated BtA-treated males was 16.67%.

## Discussion

Chemical communication systems in moths may change due to selection factors operating in the natural and the managed environment.Female *P.gossypiella* moths emitted significantly more sex pheromone when subjected to mating disruption for population control [33]. In this study, we found changes in the sex pheromone communication system in *H.armigera* under treatment by BtA.

A significant reduction in male pupal weight was observed after feeding with artificial diet containing different concentrations (from 1 µg/g to 8 µg/g) of BtA especially at the concentration of 2 µg/g (**Table 1**). However, female pupal weight significantly increased after feeding with artificial diet containing lower concentrations (from 0.5 µg/g to 2 µg/g) of BtA especially at the concentration of 2 µg/g (**Table 1**). Wanna et al. (2010) showed that mean pupal weight decreased significantly as BtA concentration increased [10]. We speculate that larvae strong enough to survive with BtA treatment and display an increased mean pupal weight may have improved fitness to contribute to subsequent generations. However, there was no evidence of correlation between pupal weight and sex pheromone titer. Similar results were achieved by Miller and Roelofs (1980), Charlton and Cardé (1982), Schal and Cardé (1987), and Löfstedt et al. (1985)[34]–[37].

The female survivors from the larvae treated with a lethal dose (LD_20_) of BtA reduced the percentage of calling while the periodicity of female survivors was similar to control females (**Figure 4**). The start calling time and peak calling timing of BtA-treated females was delayed slightly compared with control females (**Figure 4**). Female *P.xylostella* moth resistance to tebufenozide and abamectin exhibited a similar trend [22], as did *S.litura* [20] and *O.furnacalis* female survivors with larvae treated with deltamethrin [19]. Our results were also consistent with those in adult moths treated with pesticides such as *O.furnacalis* dosed with deltamethrin [18], *P.gossypiella* dosed with permethrin [12] and *T.ni* dosed with cypermethrin [17]. However, the percentage of peak calling in the *P.xylostella* female survivors with indoxacarb treatment was higher than that of control females [23]. Higher calling percent implied that females had a higher sex pheromone emitting rate. This may be one of the reasons why control females could attract the most males to mate with them (**Figure 6**). Further more, the nervous systems of surviving female moths were likely to be damaged by BtA that may have resulted in a lower calling rate.

The mean titers of Z11–16:Ald and Z9–16:Ald in BtA-treated females were increased significantly (P<0.01) compared with control females (**Table 2**). This is consistent with *S.litura* and *O.furnacalis* survivors from larvae treated with deltamethrin [19], [20]. To the contrary, abamectin resistant *P.xylostella* female moths produced less sex pheromone [22], as did Bt-treated females of *H.armigera* [29]. In addition, the mean coefficient of variations of Z11–16:Ald, Z9–16:Ald and sum extracted from BtA-treated female ovipositors were significantly higher (P<0.01) than in the controls (**Table 2**). This may imply that production of sex pheromone from BtA-treated females was not as stable as that of control females. Biosynthesis and emission of sex pheromone in lepidopteron females is regulated by their nervous systems, PBAN, PBAN-receptor, juvenile hormone, cAMP, Ca^2+^ pathway and other factors [38]–[43]. The mixed biopesticide we used in these experiments was *Bacillus thuringiensis* kurstaki and abamectin that have different targets. The Cry proteins target the insect midgut and are activated by midgut proteases. Activated toxins, interacting with the larval midgut epithelium, cause a disruption in membrane integrity and lead to insect death [44]. In contrast, abamectin acts on GABA and glutamate-gated chloride channels [45], and induces enhanced cytochrome P450 monooxygenase activity [46], [47]. Monooxygenase has a relationship with sex pheromone production [48], sometimes being involved in converting the structure and components of the sex pheromone [49]. Also, juvenile hormone is, affected by abamectin [50] and may up-regulate the level of PBAN analog protein [51] and PBAN receptors [42]. We detected that many pupae were in diapause after the larvae were treated with BtA (**Table 1**). Diapause of *H.armigera* would be broken by some multiple peptides in the FXPRL family [52]. These peptides are encoded by diapause hormone - pheromone biosynthesis activating neuropeptide gene (DH-PBAN gene) [53]. Wei et al. (2004) speculated deltamethrin may enhance the level of calcium ionophore that leads to high PBAN-like activity and higher sex pheromone production [19]. Alternatively, Xu et al. (2010) proposed the enhancement of cytochrome P450 monooxygenase activity may leads to lower production of sex pheromone [22]. We could not confirm if either mechanism led to a higher production level of sex pheromone in female survivors from larvae treated with BtA because of the more complex mechanisms operating in the mixed pesticide compared to the single pesticide. However, we assume that BtA may interfere levels of monooxygenase, juvenile hormone and other enzymes in *H.armigera* larvae, as well as the nervous system development and the PBAN pathway in female moths. Alternatively, we hypothesized that up-regulation of the DH-PBAN gene may lead to high expression of PBAN.

High sex pheromone titer did not directly translate to an increase in release rate as shown in **Figure 4** and **Table 2**. High emitting rate would increase the distance of communication [37], but the ratio of components is a more important factor [22], [54]. The mean ratio of Z11–16:Ald and Z9–16:Ald produced by BtA-treated females was shifted from 9.76 to 5.80 (**Table 2**), similar to that found in abamectin-resistant *P.xylostella* [22]. This shift of blend ratio may also lead to a lower mating rate of female survivor moths from larvae treated with BtA (**Figure 6**). However, there were still some control males and BtA-treated males that mated with BtA-treated females (**Figure 6**). Further research would be needed to verify the existence of these males with broader response to the blend ratio of sex pheromones produced by BtA treated females. The olfactory insect system is so complex [55] and selective that male moths, for example, can discriminate female-produced sex pheromones from compounds with minimal structural modifications [56]. Individual conspecific pheromone components may act as behavioral antagonists when they are emitted at excessive rates and ratios [57]. Also, a model known as “asymmetric tracking” has two steps for the evolution of sex pheromone communication systems [58]. First: males can respond to an unusual blend but still retain ability of responsiveness to the ancestral pheromone blend. The second step, known as assortative mating, occurs between females which emit a new blend or new components and males that respond exclusively to this new sex pheromone [58]. Our work identifies the possibility that the effect of BtA could result in assortative mating, although further studies are needed. Finally, a new communication system may evolve following treatment of BtA generation after generation (high resistant strain to BtA); this phenomenon was found in abamectin resistant *P.xylostella* [22]. We have conducted preliminary tests in the wind tunnel (data not shown) that show control males and BtA-treated males remain sessile at the “take-off” stand when exposed to plumes from lures containing more than 10% Z9–16:Ald. Taken together, these findings show that pheromone blend ratios are more important for mating success than are pheromone titers.

In the wind tunnel bioassays, the percentage of “OR” in BtA-treated males was significantly higher (P<0.05) than control males, however percentages of “APP” and “LA” were significantly lower (P<0.01) (**Figure 5**). This phenomenon of a lower percent response to the lure in the wind tunnel and higher production of sex pheromone was called a “compensation effect” by Delpuech et al. (2001) [59] and Wei et al. (2004) [19] and is a testable hypothesis. Abamectin resistant *P.xylostella* moths show a higher percent response to the lure and a lower production of sex pheromone, which suggests that an inverse form of compensation may be operating. We deduce that there may be a dynamic effect on the pheromone communication system that initially occurs where a dose below the threshold value for the lethal dose of pesticide has a promoting effect but a high-dose over the threshold value has an inhibiting effect. We noted that the 3% percent landing rate for BtA-treated males is very low. We suggest that BtA may damage and disturb some pathways in the development of nervous and olfactory systems in males that affect responses to blend ratios.

The importance of this is that BtA has not only been used for control of *H.armigera*, but also *Dendrolimus punctatus*, *Ceracris kiangsu*, *Pantana phyllostachysae*, *Algedonia coclesalis*, *P.xylostella* and other agricultural and forest pests [25], [27], [60]–[62]. Consumption of BtA in several provinces of China was in the hundreds of tons in 2011. We initiated using *H.armigera* as a model organism to evaluate the effect of mixed BtA biopesticide on the sex pheromone communication system and other reproductive behaviors.

The specific mechanisms for why the sex pheromone communication system changed after larvae were treated with BtA remains unclear; a better understanding of how BtA affects *H.armigera* could point us to a course of action that may include using a new ratio of lure, a more suitable dose of pesticide in the field, etc. to accommodate these changed reproductive behaviors.
